# Influence of water matrix and hydrochar properties on removal of organic and inorganic contaminants

**DOI:** 10.1007/s11356-020-09164-7

**Published:** 2020-05-26

**Authors:** Mirva Niinipuu, Magnus Bergknut, Jean-François Boily, Erik Rosenbaum, Stina Jansson

**Affiliations:** 1 Department of Chemistry, Umeå University, SE-90187 Umeå, Sweden; 2 Industrial Doctoral School, Umeå University, SE-90187 Umeå, Sweden; 3 MTC-Miljötekniskt Center AB, Dåva Energiväg 8, SE-90595 Umeå, Sweden

**Keywords:** Adsorption, Sewage sludge, Paper mill sludge, Horse manure, Hydrochars, Water treatment

## Abstract

The removal of contaminants from water using low-cost adsorbents has been widely studied, yet studies employing a realistic water matrix are still lacking. This study investigated the removal of organic compounds (trimethoprim, fluconazole, and perfluorooctanoic acid (PFOA)) and metals (As, Zn, and Cu) from landfill leachate. Additionally, tests in pure water, humic acid, and ion matrices were carried out to better understand how the water matrix affects adsorption. The hydrochars were produced from four feedstocks at three carbonization temperatures. The results show that the removal efficiencies for organic pollutants were low and metal removal by hydrochars was comparable with commercial activated carbon. The removal of all compounds from pure water was substantially lower. Tests with humic acid and ion-containing matrices could not fully explain the increased removal in the landfill leachate, which may be due to the combination of the water matrix and presence of soluble species from the hydrochars.

**Electronic supplementary material:**

The online version of this article (10.1007/s11356-020-09164-7) contains supplementary material, which is available to authorized users.

## Introduction

Sustainable feedstock materials for the production of low-cost adsorbents have been studied extensively. These low-cost adsorbents have mostly been produced via various carbonization techniques, such as pyrolysis and hydrothermal carbonization (HTC); the latter of which has recently gained widespread attention due to its potential to handle wet materials more efficiently than dry carbonization techniques. A drying step prior to carbonization is unnecessary, and HTC makes water removal after carbonization easier due to the increased hydrophobicity of the produced material and the rupture of cell walls during the HTC process (Garcia Alba et al. 2012; Pavlovic et al. 2013; Vom Eyser et al. 2015). A wide variety of precursor materials, such as manure, sewage, and paper mill sludge, food waste, municipal waste as well as wood and agricultural residues, have been studied for their suitability to HTC treatment (Oliveira et al. 2013; Alatalo et al. 2013; Huff et al. 2014; Peng et al. 2017; Weidemann et al. 2018). Carbons produced via HTC, commonly known as hydrochars, possess high densities of oxygen- and nitrogen-bearing reactive functional groups. These groups show an affinity for organic and inorganic contaminants via various interactions (e.g., hydrogen binding, dipole-dipole, dipole-induced-dipole, complexation) (Lu et al. 2012; Sun et al. 2012; Fang et al. 2014). Inorganic species present in carbon materials may promote ion exchange and the precipitation of metals (Li et al. 2017). On the other hand, the uptake of contaminants may be compromised due to the relatively low surface areas which are typical for non-activated hydrochars (Fang et al. 2018).

The removal of a wide range of organic and inorganic species by low-cost adsorbents has been extensively studied using single-component model water systems (Mohan et al. 2014). However, the behaviors of multi-component systems and the water matrix, especially under environmentally relevant concentrations, are poorly understood (Silva et al. 2018). Typically, studies on the adsorption of environmental contaminants are conducted in the mg L^−1^ range, which is orders of magnitude higher than actual concentrations in influents and therefore unrealistic (Östman et al. 2017; Silva et al. 2018). This caveat is important to consider when assessing the suitability of various materials to real-life scenarios, and the limitations of previous contaminant adsorption studies have been highlighted in recent publications (Sedlak 2018; Silva et al. 2018).

This study aimed to investigate how HTC temperature and feedstock composition affect the contaminant removal efficiency of the resulting hydrochars. A second objective was to assess how the water matrix influences adsorption. The presented research investigated the removal efficiencies of trimethoprim, fluconazole, perfluorooctanoic acid (PFOA), arsenic, zinc, and copper—all at environmentally relevant concentrations—from complicated water matrices. The removal kinetics of contaminants from a landfill leachate water matrix were investigated using hydrochars produced from four different feedstocks and carbonized at three different temperatures. Additionally, the contributions of dissolved aqueous species to the observed removal efficiencies were tested through adsorption experiments in ultrapure water as well as in aqueous solutions of humic acids and ions.

## Materials and methods

### Hydrochar material preparation

Four locally generated residue materials were selected for this study as follows: fiber sludge, biosludge, sewage sludge, and horse manure. These residues are generated in large volumes and are currently inefficiently utilized. The fiber sludge and biosludge, which represent undigested and digested sludge types, respectively, were collected from a pulp and paper mill in Sweden. The sewage sludge (anaerobically digested sludge) was sampled from a municipal wastewater treatment plant (Umeå, Sweden), and the horse manure (mixture of manure and sawdust) was collected at a local stable (Vännäs, Sweden). The feedstock materials were homogenized and mixed with water, resulting in dry material contents ranging between 10 and 22% (10%, 19%, 19%, and 22% for the biosludge, horse manure, fiber sludge, and sewage sludge, respectively). Next, approximately 600 mL of raw material was carbonized in a 1-L HTC reactor (Amar Equipments, Mumbai, India) for 2 h after the peak temperature was reached. Each material was carbonized at 180 °C, 220 °C, and 260 °C. In order to remove water-soluble species from the hydrochar surface, the materials were washed with agitation in ultrapure water in suspensions of 100 g L^−1^ for 1 h, which was followed by filtration (Munktell analytical filter paper grade 3, < 10 μm; Ahlstrom-Munksjö, Helsinki, Finland) and rinsing (three times with ca. 200 mL of ultrapure water). The hydrochar materials were thereafter dried at 105 °C overnight.

### Adsorption tests

Batch kinetic adsorption tests were conducted to determine the adsorption kinetics of the produced hydrochar materials. Landfill leachate was sampled from a hazardous waste landfill pond in northern Sweden and frozen in plastic bottles directly after sampling. The water was stored at − 18 °C before the adsorption tests. Prior to the adsorption tests, a batch of water was thawed at room temperature and filtered through a stack of glass wool, Whatman GB/B prefilter, and GF/F filter (0.7 μm). After filtering, the water was spiked with stock solutions containing trimethoprim, fluconazole, and PFOA to achieve the final concentrations of 800 ng L^−1^. The water was also spiked with solutions containing zinc(II), copper(II), and arsenic(V) (all TraceCERT**®** reference materials, Sigma Aldrich, St. Louis, MO) to obtain final concentrations of 500 μg L^−1^ for Cu and Zn and 20 μg L^−1^ for As. The spiking concentrations reflected what had previously been reported in Swedish wastewater plant influent (Östman et al. 2017). The characteristics of the filtered and spiked leachate are described in Table 1 (analyzed by Eurofins AB, Lidköping, Sweden).

**Table 1:** Leachate water matrix characteristics along with the spiked pollutant concentrations used in this study

Ion concentration	Concentration (mg L^−1^)
Iron	0.12
Calcium	190
Potassium	470
Magnesium	30
Manganese	0.4
Sodium	520
Chloride	2000
Fluoride	< 0.20
Sulfate	45
Nutrients	
Ammonium	23
Ammonium-nitrogen (NH_4_-N)	18
Nitrate (NO_3_)	< 0.44
Nitrogen bound as nitrate (NO_3_-N)	< 0.10
Nitrite (NO_2_)	< 0.0070
Nitrogen bound as nitrite (NO_2_-N)	< 0.0020
Phosphate (PO_4_)	0.13
Phosphorus, bound as phosphate (PO_4_-P)	0.043
Other	Value
pH	7.7
Conductivity	770 mS m^−1^
Hardness	34°dH
Turbidity	0.9 FNU
Chemical oxygen demand (COD-Mn)	57 mg O_2_ L^−1^
Dissolved organic carbon (DOC)	38 mg L^−1^
Color (410 nm)	14 mg Pt L^−1^
Alkalinity	250 mg HCO3 L^−1^
Spiked adsorbate concentration	
Arsenic(V)	20 μg L^−1^
Copper(II)	500 μg L^−1^
Zink(II)	500 μg L^−1^
Trimethoprim	800 ng L^−1^
Fluconazole	800 ng L^−1^
PFOA	800 ng L^−1^

Kinetic adsorption tests of the spiked leachate water were performed for all twelve produced hydrochars, and contaminant removal was measured over 3, 7, 15, 30, and 60 min. All the tests were performed in triplicates. Adsorption tests were initiated by weighing 0.500 g of each hydrochar and mixing it with100 mL of leachate. At each time point, samples—ca. 5 mL for organic analysis, 50 mL for inorganic analysis, and 20 mL for pH measurement—were collected with a plastic syringe and filtered through a 0.45-μm filter. For time point *t = 0*, the leachate was sampled and filtered at the same time as each batch of kinetics tests was initiated. The samples for organic analysis were weighed, after which isotopically labeled internal standards (D_4_ fluconazole, ^13^C,D_3_ trimethoprim, and ^13^C_2_ PFOA) were added. The pH at each time point was recorded directly after the adsorption tests with a PHM290 pH-meter (Radiometer Analytical SAS, Villeurbanne Cedex, France). Samples were stored at − 18 °C prior to organic and metal analyses. Additionally, the 60-min time point included a comparison with a commercial granular activated carbon (Aquasorb 2000 20 × 40 mesh). The possible adsorption of analytes to the test tube walls was controlled for by shaking the spiked water samples for 60 min. In addition, seven blank samples (consisting of distilled water) were shaken for 60 min.

Adsorption tests of the sewage sludge and horse manure hydrochars that had been carbonized at 220 °C were performed with distilled water, humic acid, and salt solutions (concentrations specified later) to investigate how the natural water matrix influences removal efficiency. In all cases, the pH was adjusted to 7.7 with 0.1 M and 0.5-M HCl and NaOH solutions to simulate the leachate pH. Furthermore, each solution was spiked with the same concentrations of analytes as had been used in leachate water tests. The humic acid stock solution was prepared in a 0.05-M NaOH solution and filtered through a Whatman GF/F filter (0.7 μm). The humic acid concentration was 86 mg L^−1^, which corresponds to the dissolved organic carbon (DOC) concentration in the leachate water matrix (38 mg L^−1^) when considering the 20% ash content of humic acid (reported by the supplier) and assuming that the carbon content of humic acid is ~ 55% (Allard 2006). A model solution containing the most abundant ions in leachate water was prepared by dissolving 1.29 g L^−1^ NaCl, 0.895 g L^−1^ KCl, 0.527 g L^−1^ CaCl_2_, and 0.750 g L^−1^ MgSO_4_ in ultrapure water.

The metal analyses were outsourced to Eurofins AB (Lidköping, Sweden), where the samples (including possible precipitations) were digested with nitric acid and analyzed by inductively coupled plasma mass spectroscopy (ICP-MS; ISO 15587-2:2002 and ISO 17294-2:2016). Organic compounds were identified using a Thermo Quantiva mass spectrometer equipped with an on-line SPE column and a Hypersil Gold (50 × 2.1 mm, 3 μm particles size) analytic column (Thermo Scientific, Waltham, MA) and operated in positive (trimethoprim and fluconazole) and negative (PFOA) ion modes HESI (heated electrospray ionization). The limits of quantification for all analytes are shown in Table S1 (Supporting information).

The removal efficiency (%) was calculated as follows:

1 $$ removal\ efficiency\ \left(\%\right)=\left(\frac{C_{\mathrm{i}}-{C}_{\mathrm{e}}}{C_{\mathrm{i}}}\right)\times 100\% $$

where *C*_i_ and *C*_e_ (μl L^−1^ and ng L^−1^) denote the initial concentration in water and the concentration at equilibrium (after 60 min), respectively.

The adsorption capacity, *q*_t_ (μl L^−1^ and ng L^−1^), at time point *t* (min) was calculated according to:

2 $$ {q}_{\mathrm{t}}=\frac{\left({C}_{\mathrm{i}}-{C}_{\mathrm{t}}\right)}{m}V $$

where *C*_t_ (μl L^−1^ and ng L^−1^) is the concentration in water at time point *t*, *m* (g) is the mass of the adsorbent, and *V* (L) is the volume of the solution. Thereafter, the obtained data was fitted to non-linear pseudo-first-order and pseudo-second-order kinetic models. The non-linear Lagergren pseudo-first order model (PFO) is expressed as (Lagergren 1898):

3 $$ {q}_{\mathrm{t}}={q}_{\mathrm{e}}\left(1-{e}^{-{\mathrm{k}}_1\mathrm{t}}\right) $$

where *q*_t_ (ng g^−1^ or μg g^−1^) is the adsorption capacity at time = *t*, *q*_e_ (ng g^−1^ or μg g^−1^) is the adsorption capacity at equilibrium, and k_1_ (min^−1^) is the pseudo-first order rate constant. The non-linear form of the pseudo-second order model (PSO) is expressed as (Blanchard et al. 1984):

4 $$ {q}_{\mathrm{t}}=\frac{q_{\mathrm{e}}^2{k}_2t}{1+{q}_{\mathrm{e}}{k}_2t} $$

where *k*_2_ (g ng^−1^ min^−1^ or g μg^−1^ min^−1^) is the pseudo-first order rate constant.

### Principal component analysis

Principal component analysis (PCA) was conducted to visualize the differences in hydrochar properties and removal efficiencies (data reported in a previous study (data reported by Niinipuu et al. (2020))). The model included the following material properties: X-ray photoelectron spectroscopy (XPS) data with elemental composition as well as carbon, oxygen, and nitrogen functionalities; specific surface area; char pH; and methylene blue maximum adsorption capacity. Additionally, the removal efficiency of each compound and the total removal efficiency were included in the model. SIMCA-P software (version 14, Umetrics AB, Umeå, Sweden) was used for the PCA analysis. The data were mean centered and scaled to unit variance.

## Results and discussion

### Landfill leachate matrix

After 60 min, the sewage sludge hydrochars showed higher removal efficiencies overall (25–42%, average removal efficiencies of all compounds) as compared with the other studied hydrochars, all of which showed similar removal efficiencies (11–32%) (Table 2). The corresponding value for the commercial activated carbon was 66%. The removal efficiency was substantially decreased in hydrochars produced at the highest temperature, while the hydrochars produced at 180 °C and 220 °C showed similar removal efficiencies. These results suggest that both treatment temperature and choice of feedstock affect the adsorption efficiencies of hydrochars for a given contaminant.

**Table 2:** The average removal efficiencies for the studied materials

Temperature			
Feedstock	180 °C	220 °C	260 °C
Horse manure	26 ± 8%	29 ± 7%	11 ± 14%
Fiber sludge	32 ± 6%	29 ± 6%	14 ± 10%
Sewage sludge	42 ± 6%	36 ± 7%	25 ± 7%
Biosludge	26 ± 7%	31 ± 7%	18 ± 10%

**Fig. 1:**
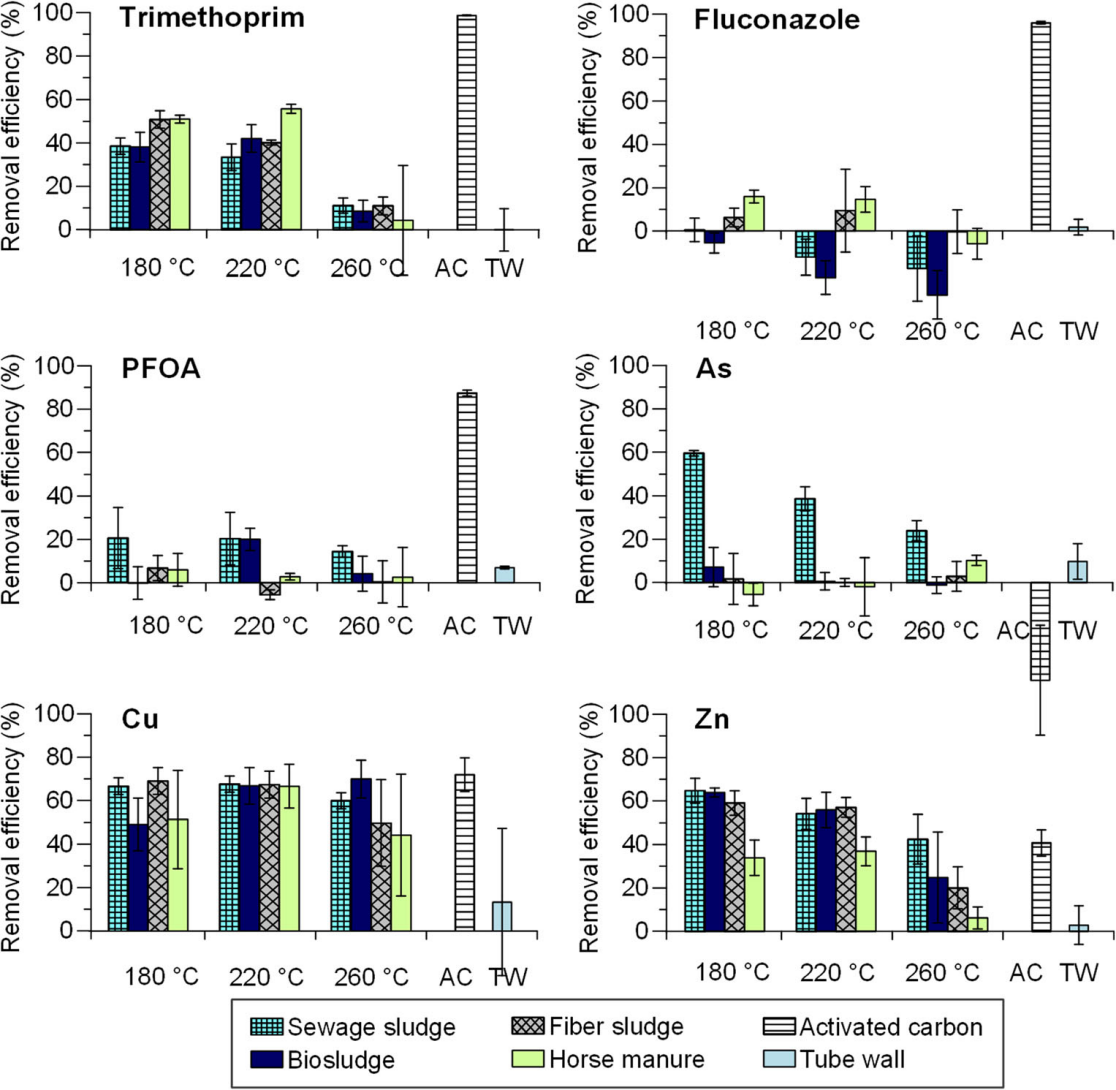
The removal efficiencies of hydrochars, commercial activated carbon, and tube wall, in leachate water after 60 min of adsorption. The error bars indicate ± 1 standard deviation

The results show that, in general, the removal of the studied compounds decreased as HTC treatment temperature increased. This was most evident for trimethoprim and Zn(II) in the case of horse manure and fiber sludge hydrochars and for As(V) in the case of sewage sludge hydrochars (Fig. 1). The removal efficiencies observed for all compounds except Zn(II) and As(V) were similar regardless of hydrochar feedstock material. Horse manure hydrochars clearly had the lowest affinity for Zn(II) when compared with the other tested materials, while sewage sludge hydrochars were the only materials able to remove As(V). The removal capacity of the sewage sludge hydrochars was likely due to the presence of iron species in these materials, which are able to precipitate As or sorb As onto free iron (hydr)oxides (Roberts et al. 2004; Escudero et al. 2009). However, the removal efficiency decreased as carbonization temperature increased while the iron content remained constant in all the materials. This suggests that the presence of other functionalities (e.g., oxygen functionalities, which could also be expected to decline with increasing carbonization temperature) contribute to the removal of As as well.

The removal of organic compounds by hydrochars was generally low (0–20% for fluconazole and PFOA, and 4–56% for trimethoprim) when compared with the removal by activated carbon (87–99%). On the other hand, most of the studied hydrochars removed Cu(II) equally well and Zn(II) more efficiently than the activated carbon. These differences in affinity for various adsorbate compounds suggest that different surface features among the hydrochars may affect the removal mechanisms. Hydrophobic organic molecules preferentially bind to activated carbon surfaces by hydrophobic interactions and pore filling (Álvarez-Torrellas et al. 2015; Tran et al. 2017), while surfaces rich in oxygen- and nitrogen-containing groups found on hydrochars may adsorb metal ions to form dense water hydration shells that hinder the interactions with neutral molecules and the surfaces (Cao et al. 2009; Kong et al. 2011). Hydrochars produced from digested sludge chars (i.e., sewage sludge and biosludge) removed PFOA and metals more efficiently than the hydrochars produced from fiber sludge and horse manure.

The generally low removal efficiencies observed for the studied hydrochars may be partly explained by the low starting concentrations in the studied system simulating real wastewater concentrations. Very few studies have been conducted using such low concentrations (ng–μg L^−1^); instead, most previous studies have employed mg L^−1^ concentrations (Silva et al. 2018). The removal efficiencies for fluconazole and trimethoprim were similar to what was reported in our previous study, in which horse manure hydrochar removed ~ 20% of the fluconazole and ~ 25% of the trimethoprim from ultrapure water with a starting concentration of 10 μg L^−1^ (Weidemann et al. 2018). In light of the relatively high methylene blue maximum adsorption capacities (as high as 68 mg L^−1^) that were reported for the tested materials in our previous study (Niinipuu et al. 2020), the removal efficiencies presented in this paper seem very low. These decreased removal efficiencies at low contaminant concentrations may be attributed to a resistance in the molecular transfer between aqueous and solid phases due to weak concentration gradients in the solution, as was previously suggested by Li et al. (2018) This may have direct implications on using hydrochars to remove contaminants that are present at low concentrations (ng L^−1^ to μg L^−1^ range) in wastewater. On the other hand, methylene blue may have higher affinity to the studied hydrochars as compared with the compounds included in this study.

The pH in most of the adsorption kinetics tests decreased during the course of the experiments, which was due to different pH values for the tested hydrochars. Furthermore, cations release protons into the solution when binding to oxygen functionalities (such as hydroxyls and carboxyl acids), and this also decreases the pH. The smallest change in pH was observed for fiber sludge hydrochars, which showed pH values between 7.5 and 7.8 after 60 min (Fig. S1). The pH values after adsorption for horse manure, biosludge, and sewage sludge chars generated at 260 °C were a few tenths higher (7.3–7.6) than those of the corresponding hydrochars generated at 180 °C and 220 °C (pH 7.1–7.3). These pH values are very close to the pKa of trimethoprim (7.12), at which trimethoprim shifts to a positively charged state. This could be expected to affect the removal mechanism. Additionally, the lower pH may increase the hydrochar surface charge, and therefore alter how the surface functional groups interact with polar molecules and ions. However, the removal efficiencies of fiber sludge chars did not noticeably differ from what was observed for the other hydrochars, which suggests that this small decrease in pH did not greatly affect removal efficiency.

**Fig. 2:**
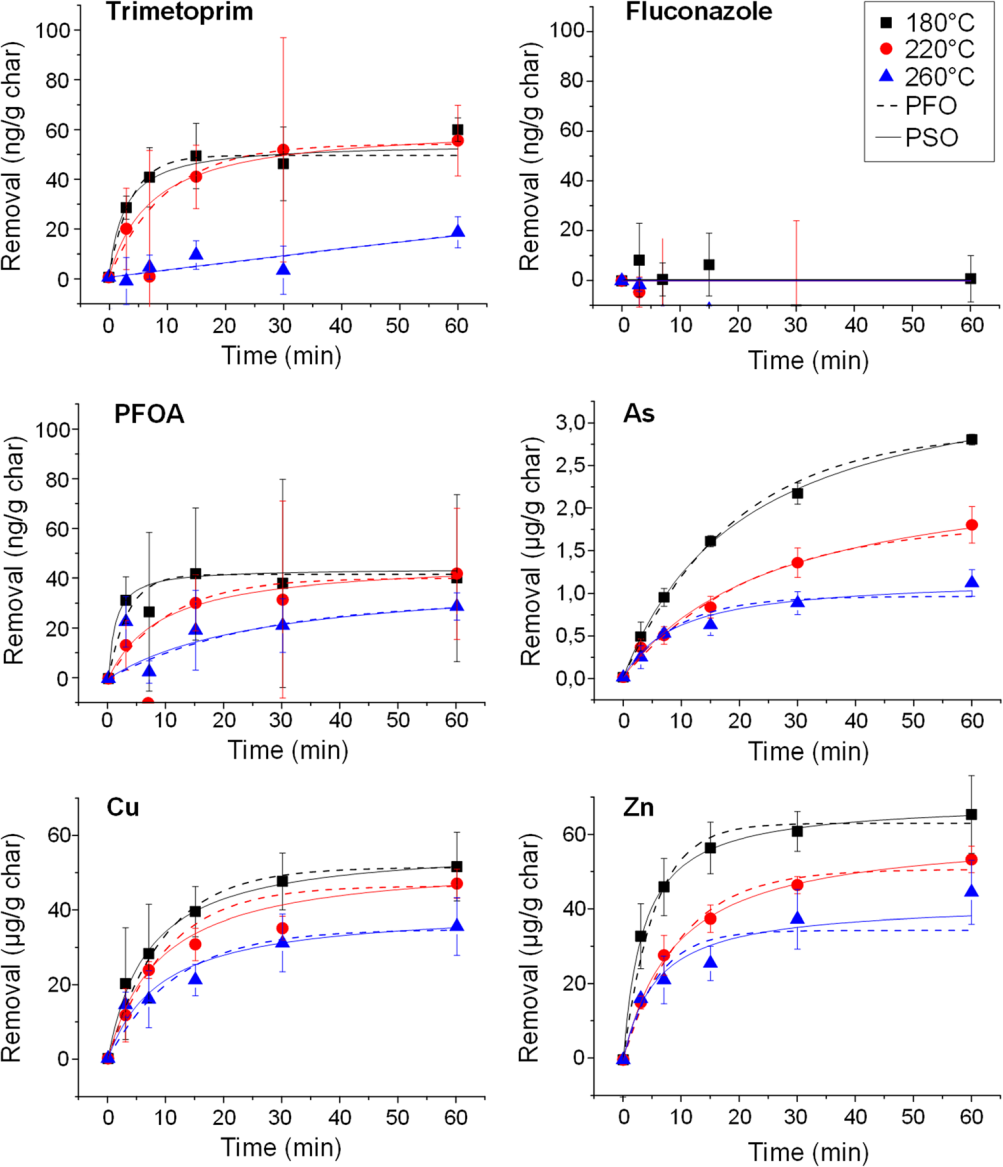
Removal kinetics for various contaminants in landfill leachate using sewage sludge hydrochars representing the entire temperature series

In general, equilibrium was reached within 30 min of contact time, but substantially slower kinetics were also observed. For instance, when sewage sludge chars were tested for As removal, the equilibrium was not entirely reached even after 1 h of contact time (Fig. 2, with the results for all other materials presented in Figs. S2–S4). Pseudo first- and second-order kinetic models were fitted to the data (model fits are presented in Supplementary Information Table S2). The removal efficiencies of fluconazole and PFOA were low or nonexistent, and therefore, kinetic models did not show a sufficient fit, while good model fits (R^2^ > 0.90 for 75% of cases) were obtained for Cu, Zn, and trimethoprim removal kinetics. The results of hydrochars prepared at 260 °C showed the poorest fit to the models, which is likely explained by the high variance between measured time points in combination with low removal efficiencies. In general, the removal of the studied compounds was better described by the pseudo-second-order model than the pseudo-first-order model.

### The influence of the water matrix

To further explore how the water matrix affects the removal efficiencies of the studied contaminants, tests in pure water as well as solutions containing humic acid and ions were carried out. A solution that included both humic acid and ions was also prepared, but agglomerates immediately formed in solution. These agglomerates severely obstructed the collection of water and possibly also contaminant retention, which was not observed in the landfill leachate matrix.

**Fig. 3:**
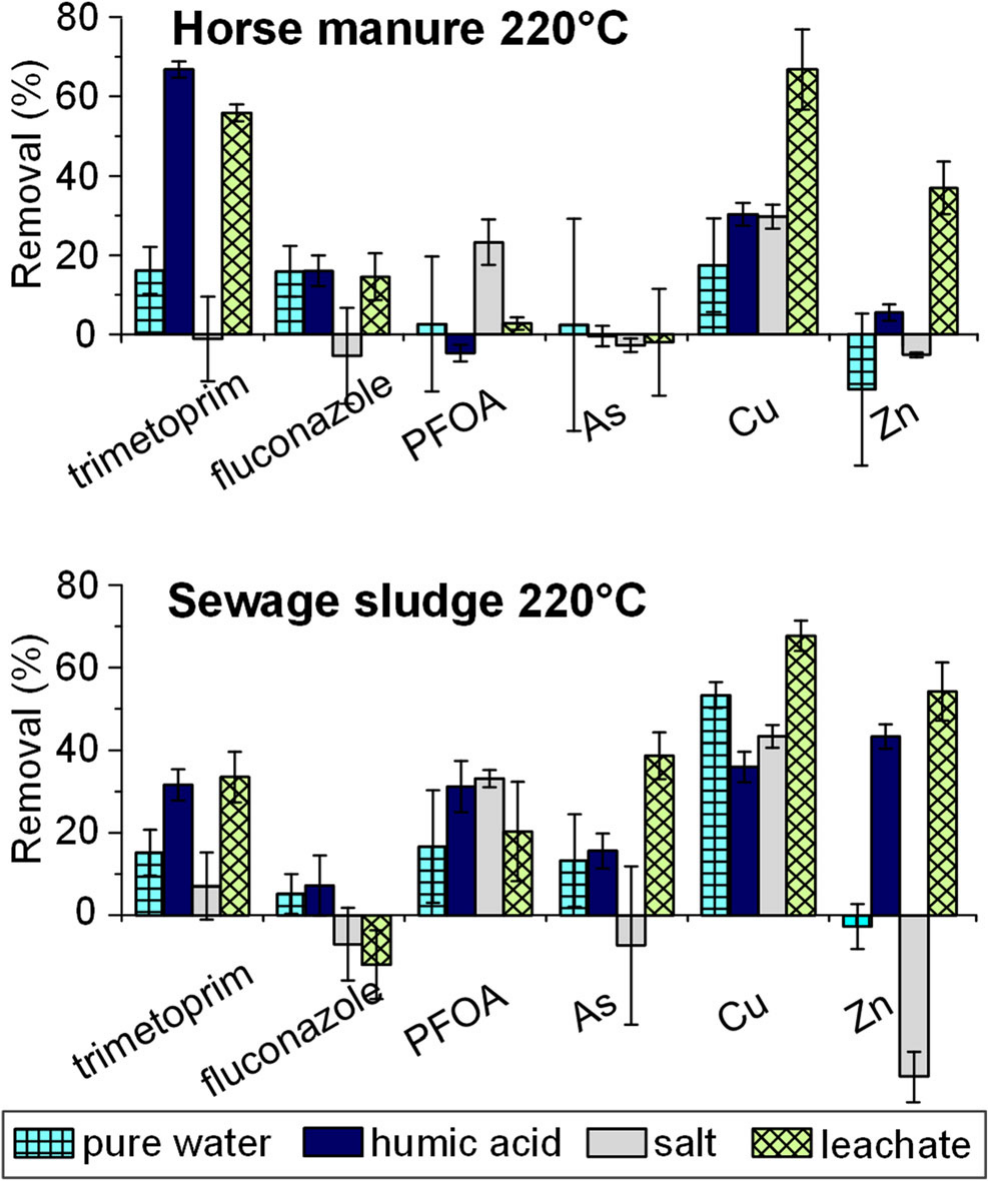
Differences in the removal efficiencies of compounds between pure water, humic acid water, ion water, and landfill leachate matrices

All of the contaminants showed drastically lower removal efficiencies in the pure water matrix as compared with the leachate water (Fig. 3). The presence of humic acid led to an increase in the removal of trimethoprim and Zn, which suggests that dissolved organic carbon in water promotes the removal of these compounds. A previous study has shown that the presence of DOC in water increases metal adsorption by increasing metal-ion complexation in solution, whereas the removal of atrazine decreases due to saturation of the adsorbent surface and blocking of adsorbent pores (Zhou et al. 2015). Other studies have found that the removal efficiencies for caffeine and triclosan decrease when DOC is present in solution (Liu et al. 2014; Álvarez-Torrellas et al. 2015). The type of DOC also impacts adsorption. For example, Bui et al. showed different adsorption efficiencies with fulvic acid and humic acid matrices when using silica to adsorb pharmaceuticals (Bui and Choi 2010). In this study, humic acid may not have been a realistic model for simulating DOC in landfill leachate since the model solution differed noticeably (e.g., agglomerate formation, color, etc.) from the leachate water. Also, DOC content in the water most likely increased during the adsorption tests due to the leaching of DOC from hydrochar, as a color increase was observed after the adsorption tests (Fang et al. 2018). Therefore, it is not likely that these substances saturated the hydrochar surface, but instead exposed more of the surface. Decreased adsorption in the presence of DOC may therefore be explained by the adsorption of contaminants to DOC (instead of hydrochar), which means that the contaminants stay in the water (Kozyatnyk et al. 2015, 2016). In contrast, if agglomerates form, for instance, due to increasing DOC concentrations and/or the presence of salts, the adsorbates may be removed with the agglomerates during filtering. This may have contributed to the removal efficiency observed for landfill leachate water.

Adsorption tests performed in the ion water matrix showed only the removal of PFOA and Cu. Sewage sludge char even seemed to leach Zn, which may be explained by ion exchange between the hydrochar and ions in the water. Di- and trivalent ions have been shown to increase the removal efficiency by acting as bridges between negative surface groups and negative adsorbates (Bui and Choi 2010); this may explain the slight improvement in PFOA removal observed in ion-containing water. On the other hand, ions in solution may compete with the adsorbate compounds and thereby counteract adsorption (Han et al. 2007; Yamauchi et al. 2014; Yang and Jiang 2014). Previous research has shown that the ionic strength of a solution influences the adsorption of pharmaceuticals to silica and can either increase, decrease, or have no effect on adsorption (Bui and Choi 2010).

Another factor that possibly affected adsorption in this study was pH, which drastically decreased following the addition of hydrochars to the model water. The decrease was more dramatic in the pure water matrix and ion matrix (final pH 4.0–4.9) and DOC matrix (4.9–6.5) when compared with the landfill leachate (7.1–7.6), which clearly had the highest buffering capacity. This alters the surface charge, making it less negative, which in turn would likely decrease the removal of cations due to lower electrostatic attraction (or increased repulsion if surface charge is positive) between cations and the char surface. This would explain the lower removal efficiencies of Zn, Cu, and trimethoprim (which is positively charged below pH 7.12) observed in the pure water, ion, and DOC matrices.

### Multivariate analysis

**Fig. 4:**
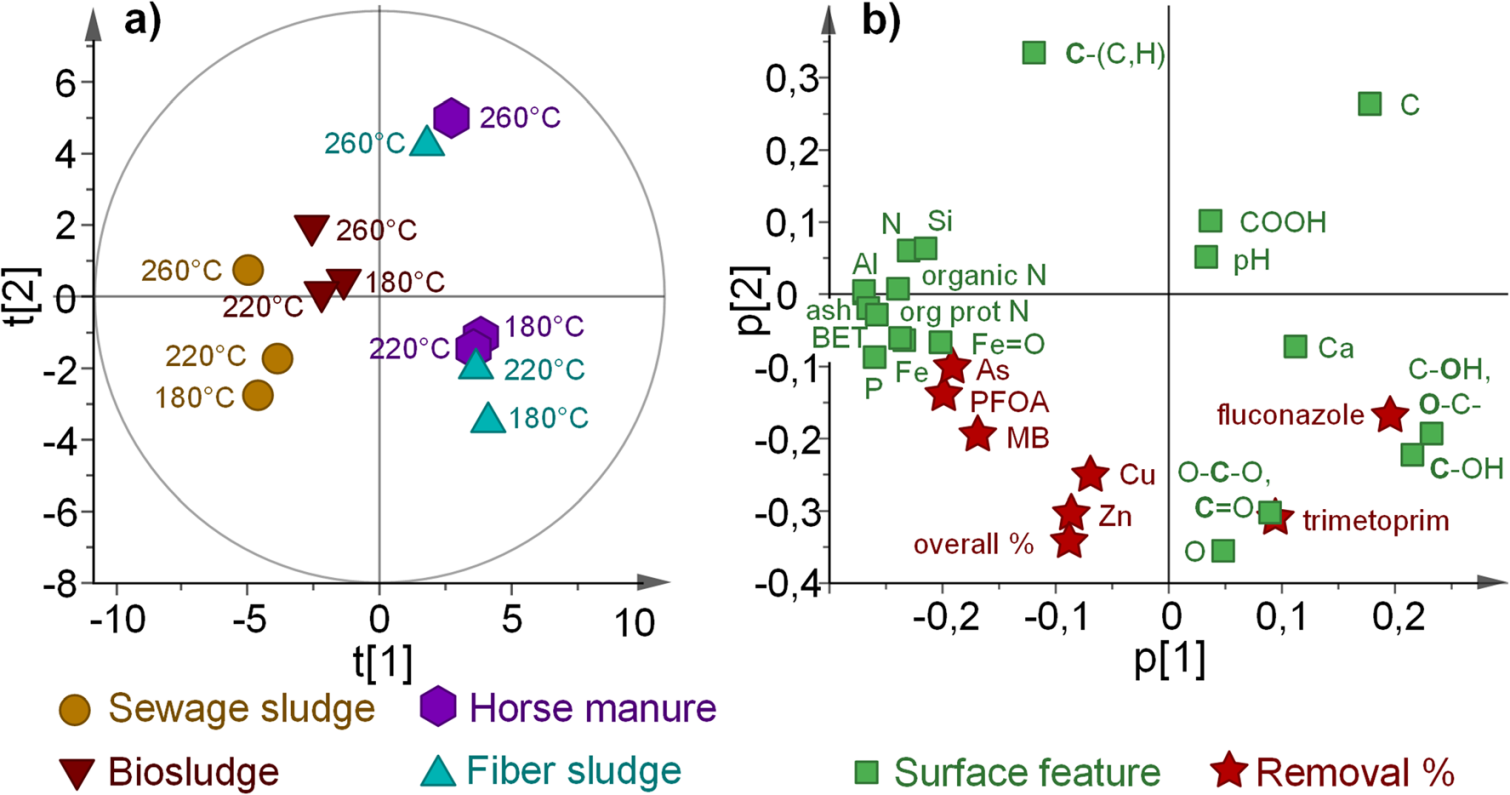
Scores (**a**) and loadings (**b**) plots for a PCA model of the surface properties and removal efficiencies of the studied hydrochars

A multivariate analysis was performed to further study the properties of the hydrochars (Niinipuu et al. 2020) and their abilities to remove the studied compounds. A principal component analysis (PCA) model was created using surface composition data from the XPS analysis and adsorption efficiencies, resulting in a model with two model components and *R*^2^ = 0.714 and *Q*^2^ = 0.444. The model clearly grouped the observations with regard to feedstock type and carbonization temperature (Fig. 4a). The first principal component (PC1), which showed 22.8% predictive ability and described 45.9% of the variation in the data, separated the feedstock materials, whereas the second principal component (PC2), which showed 27.9% predictive ability and described 25.5% of the variation, separated the carbonization temperature. The distributions seen in the scores plot (Fig. 4a) are explained by the loadings plot (Fig. 4b), which shows the variables associated with the observations in the scores plot. It was observed that mineral concentrations (such as Fe, Al, Si) and BET surface areas were associated with digested sludge types, which are grouped on the left side of the scores plot. A high content of single- and double-bonded oxygen functionalities were associated with fiber sludge and horse manure, both of which are located at the right side of the scores plot (Fig. 4a). The distribution along the second principal component was governed by carbonization temperature, and the distinction between the highest and lowest carbonization temperatures was especially clear for fiber sludge and horse manure. Both of these materials underwent substantial changes in composition at the highest carbonization temperature (i.e., decomposition of cellulosic structures).

The removal efficiency of the studied contaminants was located at the lower part of the loadings plot, which indicates that materials on the bottom of the diagram had higher removal efficiencies for the tested contaminants than materials located on the top of the plot. Trimethoprim and fluconazole removals were mainly associated with fiber sludge and horse manure, while the digested sludge types were more associated with the removal of the rest of the adsorbates. Also, the removal efficiency may be associated with other variables located close the adsorbates in Fig. 4b. For instance, a high mineral concentration may promote As removal while a high content of oxygen functionalities was associated with the removal of trimethoprim and fluconazole, but on the other hand, some variations of this may be caused by co-variation between the surface features. These possible relationships can be explained by different removal mechanisms, such as hydrogen bonding with oxygen functional groups or electrostatic interactions with minerals. The model suggests however that increasing the total carbon content and/or graphitic/aromatic carbon content would decrease the removal of the tested compounds by the studied hydrochars, which suggests that hydrophobic interactions are not associated with the removal of any of the compounds by the studied material types.

## Conclusions

In this study, the ability of hydrochars—a class of low-cost adsorbents—to remove metal ions and organic compounds from a leachate water matrix was tested at ng–μg L^−1^ concentrations. This study adds to the current knowledge by using low starting concentrations (ng–μg L^−1^), which further complicates the assessment of removal efficiencies and highlights low concentrations when estimating the applicability of these materials in real-life scenarios. The results show that for the hydrochars studied, metal cations were removed more efficiently than organic compounds. Furthermore, most of the compounds demonstrated relatively fast removal kinetics, which is advantageous for most water treatment systems (such as dynamic systems) as they are typically limited by residence time in the adsorbent column. In addition, the water matrix was shown to exert a substantial effect on removal efficiency, with all of the adsorbates showing lower removal efficiencies from ultrapure water. Modifying the ultrapure water by adding common ions or DOC could only account for some of the observed differences, while a majority of the increase in removal efficiency observed in landfill leachate could not be explained by a single matrix type. A real water matrix introduces a large degree of complexity to the removal processes, which should be addressed in future studies. The results indicate that there is a possible connection between As removal and the presence of Fe-species in the matrix. This could have commercial value and serve as a basis for enhancing low-cost hydrochars with certain properties to target specific compounds.

## Electronic supplementary material

ESM 1 (PDF 546 kb)
