# Supplementary material for: Influence of water matrix and hydrochar properties on removal of organic and inorganic contaminants
Source: Environ Sci Pollut Res Int. 2020 May 26;27(24):30333–41. doi: 10.1007/s11356-020-09164-7 (PMC7378115; doi:10.1007/s11356-020-09164-7)
Supplement: Supplementary file 1 — (PDF 546 kb) [file 11356_2020_9164_MOESM1_ESM.pdf]

Supporting information

Influence of water matrix and hydrochar properties on removal of organic and inorganic contaminants

Mirva Niinipuu [†‡], Magnus Bergknut [§], Jean-François Boily [†], Erik Rosenbaum [§], Stina Jansson [†]*

[†] Department of Chemistry, Umeå University, SE-90187 Umeå, Sweden

[‡] Industrial Doctoral School, Umeå University, SE-90187 Umeå, Sweden

[§] MTC-Miljötekniskt Center AB, Dåva Energiväg 8, SE-90595 Umeå, Sweden

* Corresponding author: stina.jansson@umu.se

Table S1. Limit of quantification (LOQ) for the analytes. For LC-MS, the LOQ is based on the standard deviation of the response and slope.

Analyte	Unit	LOQ	Method
Cu	µg/l	1	ICP-MS
Zn	µg/l	5	ICP-MS
As	µg/l	0.5	ICP-MS
Fluconazole	ng/l	1	LC-MS
Trimethoprim	ng/l	5	LC-MS
PFOA	ng/l	30	LC-MS

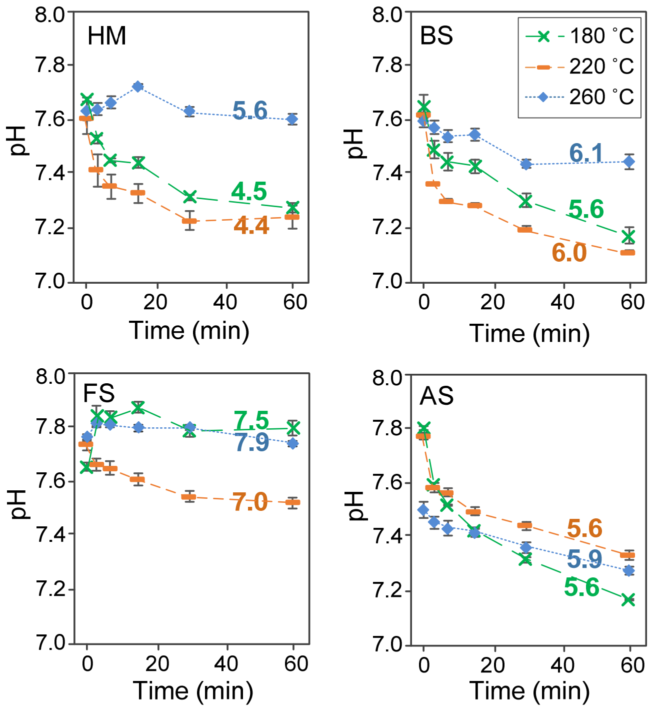

Figure S1. pH change in the leachate water during the adsorption tests. The hydrochar pH is given next to each the data series. Horse manure, biosludge, fibersludge and sewage sludge are denoted HM, BS, FS, and AS, respectively.

Table S2. Pseudo first order (PFO) and pseudo-second order model (PSO) parameters, q_e (ng/g for organic compounds and μg/g for metals), k_1 (min^{-1}), k_2 (g ng^{-1} min^{-1} or g $μg^{-1}$ min^{-1}) and R^2. Models with insufficient fit (<0.75) and unrealistic fits (nearly linear) are denoted with - and *, respectively.

		PFO			PSO				PFO			PSO		
**Trimethoprim**		q_e	k_1	R^2	q_e	k_2	R^2	**Arsenic**	q_e	k_1	R^2	q_e	k_2	R^2
Horse manure	180	83.4	0.109	0.951	94.5	0.00150	0.981		-	-	-	-	-	-
	220	72.6	0.275	0.875	81.9	0.00387	0.944		-	-	-	-	-	-
	260	8.68	0.077	0.813	10.9	0.00783	0.799		-	-	-	-	-	-
Biosludge	180	*	*	*	*	*	*		-	-	-	-	-	-
	220	63.9	0.136	1.000	74.2	0.00219	0.994		-	-	-	-	-	-
	260	19.7	0.039	0.772	31.6	0.000761	0.760		-	-	-	-	-	-
Sewage sludge	180	48.9	0.266	0.928	53.8	0.00690	0.941		2.87	0.0526	0.997	3.71	0.0128	1.000
	220	53.5	0.101	0.912	61.0	0.00219	0.946		1.78	0.0456	0.985	2.50	0.0150	0.989
	260	-	-	-	-	-	-		0.948	0.109	0.972	1.16	0.0949	0.986
Fiber sludge	180	70.2	0.156	0.815	78.9	0.00261	0.917		-	-	-	-	-	-
	220	60.5	0.231	0.994	63.7	0.00613	0.997		-	-	-	-	-	-
	260	17.7	0.240	0.927	18.2	0.0308	0.890		-	-	-	-	-	-
**Fluconazole**								**Copper**						
Horse manure	180	*	*	*	*	*	*		33.2	0.204	0.756	-	-	-
	220	21.9	0.1615	0.974	25.1	0.00766	0.983		33.7	0.558	0.897	36.57	0.0272	0.955
	260	-	-	-	-	-	-		31.3	0.183	0.859	-	-	-
Biosludge	180	-	-	-	-	-	-		37.7	0.290	0.998	40.68	0.01076	0.996
	220	-	-	-	-	-	-		43.3	0.213	0.880	46.26	0.00747	0.980
	260	-	-	-	-	-	-		43.0	0.147	0.921	50.52	0.00351	0.960
Sewage sludge	180	-	-	-	-	-	-		51.2	0.105	0.977	57.26	0.00258	0.996
	220	-	-	-	-	-	-		46.2	0.101	0.976	53.24	0.00212	0.991
	260	-	-	-	-	-	-		34.7	0.085	0.934	40.43	0.00270	0.966
Fiber sludge	180	-	-	-	-	-	-		48.8	0.260	0.978	52.95	0.00858	0.990
	220	-	-	-	-	-	-		48.1	0.208	0.901	51.43	0.00656	0.968
	260	-	-	-	-	-	-		27.1	0.365	0.984	28.98	0.0215	0.998
**PFOA**								**Zink**						
Horse manure	180	-	-	-	-	-	-		35.8	0.194	0.976	39.28	0.00743	0.983
	220	15.8	9.963	0.797	14.1	1.00	0.781		38.0	0.490	0.969	39.72	0.0299	0.969
	260	-	-	-	-	-	-		10.9	0.0566	0.882	16.02	0.00249	0.867
Biosludge	180	-	-	-	-	-	-		63.3	0.171	0.989	70.57	0.00294	0.998
	220	37.8	0.143	0.931	43.9	0.00378	0.931		55.4	0.504	0.998	57.99	0.0178	1.000
	260	-	-	-	-	-	-		28.4	0.166	0.992	33.45	0.00581	0.992
Sewage sludge	180	41.9	0.301	0.786	-	-	-		63.5	0.187	0.951	69.29	0.00423	0.998
	220	40.4	0.0971	0.904	47.5	0.00229	0.916		51.1	0.103	0.991	61.20	0.00184	0.999
	260	-	-	-	-	-	-		34.8	0.174	0.920	42.20	0.00424	0.960
Fiber sludge	180	-	-	-	-	-	-		55.7	0.465	0.989	58.74	0.0148	0.996
	220	-	-	-	-	-	-		54.0	0.595	0.987	56.62	0.0227	0.997
	260	-	-	-	-	-	-		20.3	0.176	0.849	22.43	0.0122	0.915

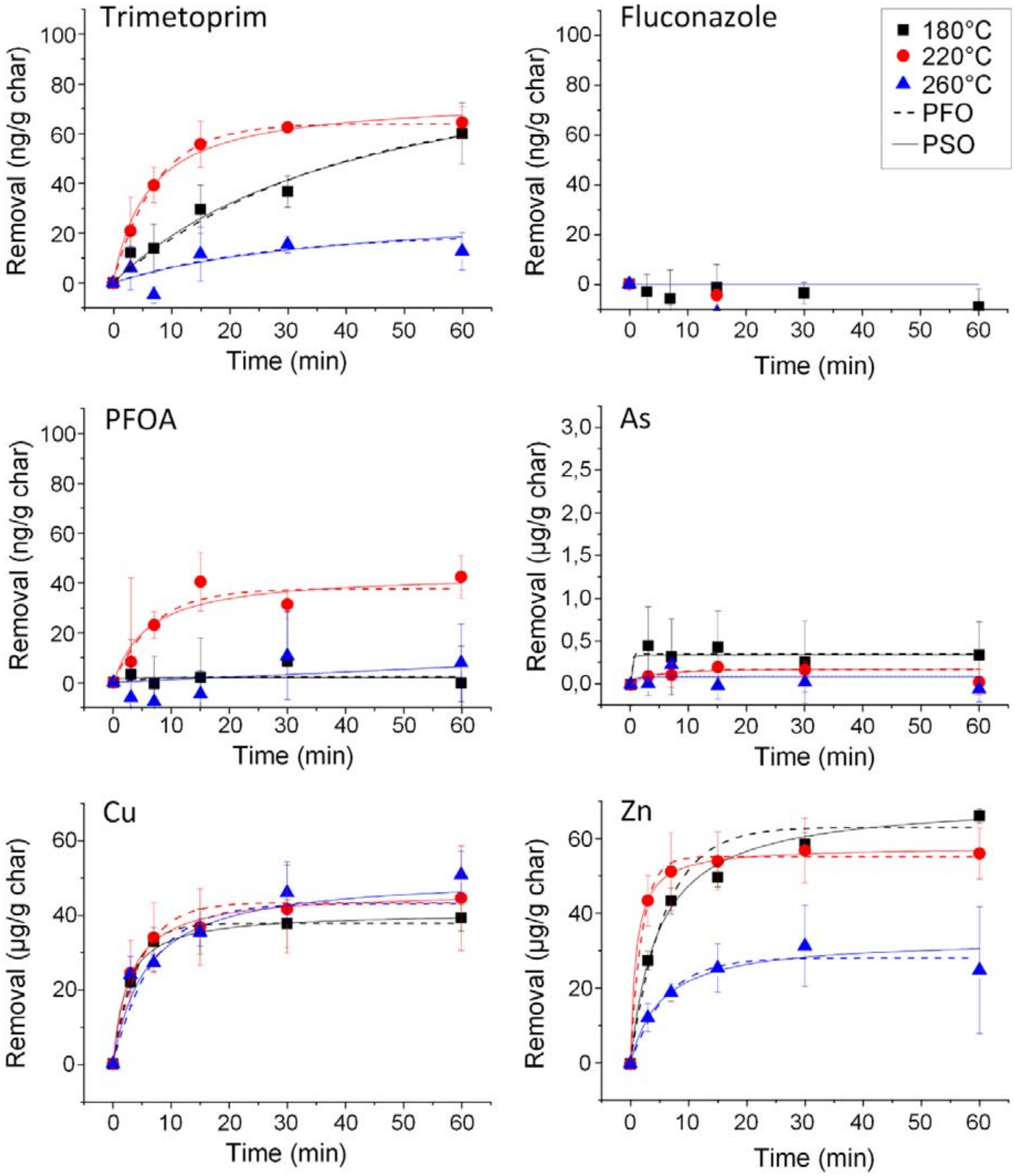

Figure S2. Removal kinetics of the studied contaminants in the landfill leachate using biosludge hydrochars from the entire temperature series.

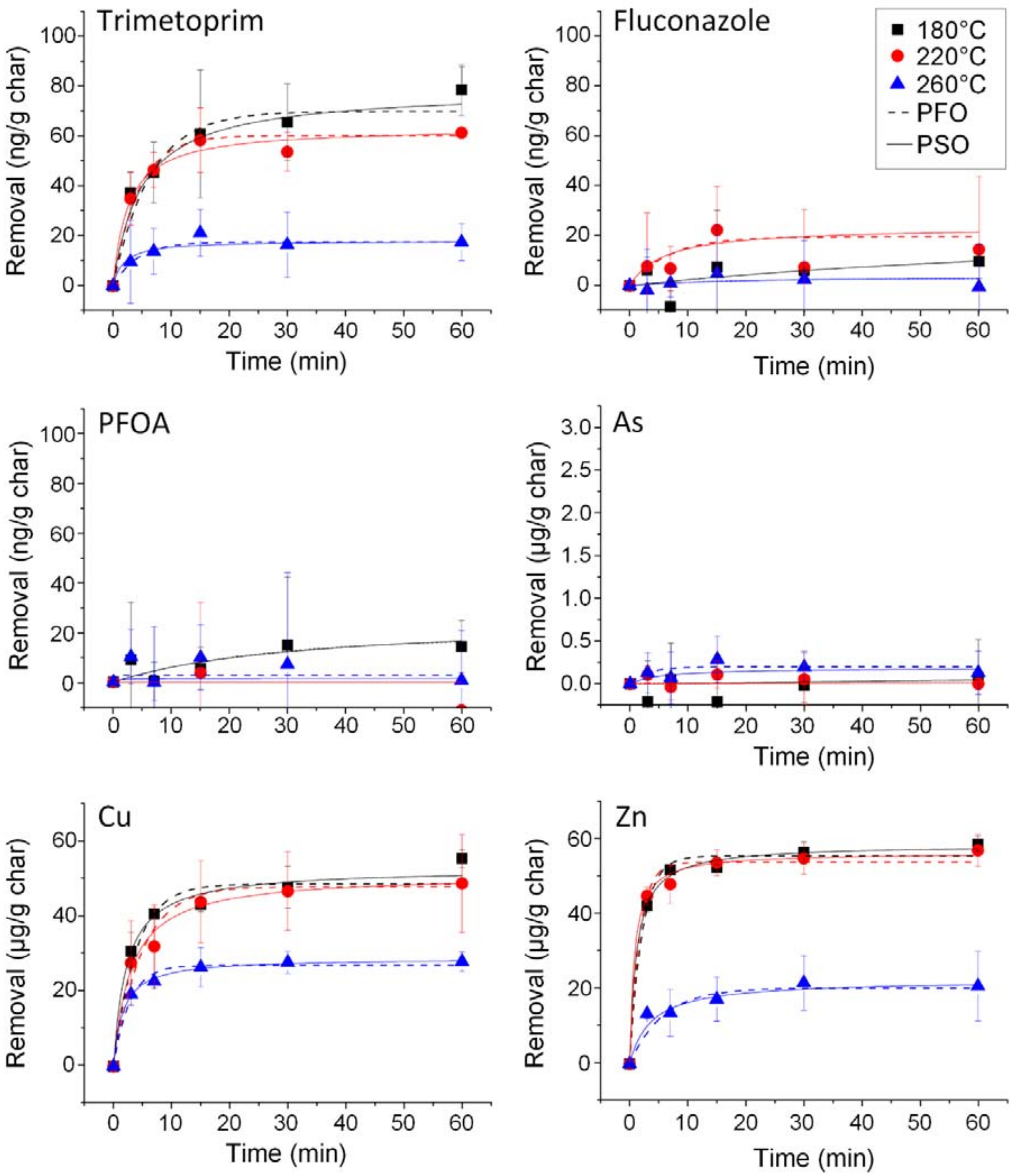

Figure S3. Removal kinetics of the studied compounds from landfill leachate on fiber sludge temperature series.

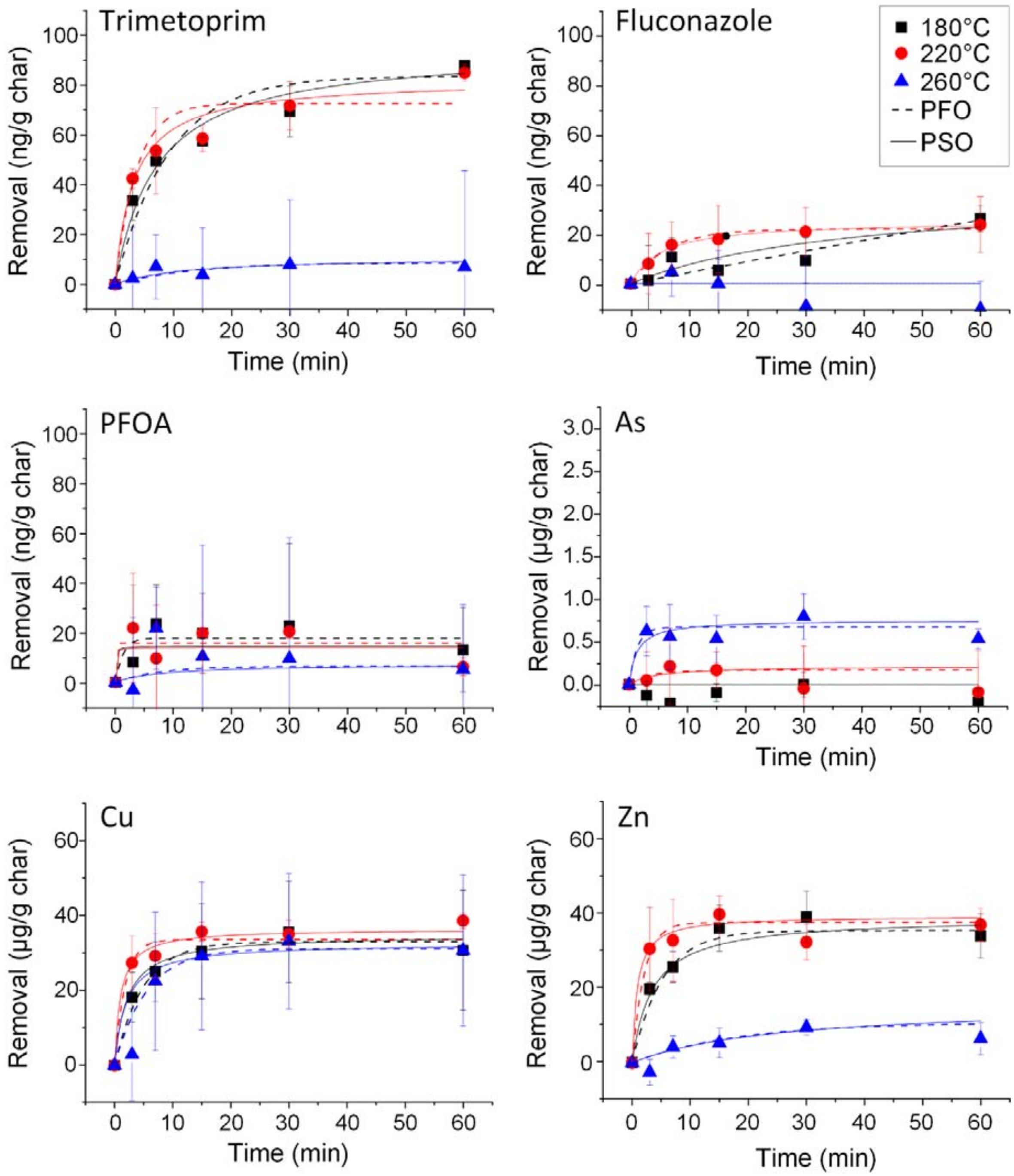

Figure S4. Removal kinetics of the studied compounds from landfill leachate on horse manure temperature series.

Table S3. Pseudo first order (PFO) and pseudo-second order model parameters for studied compounds showed in general very poor fits, likely due to the low removals of the compounds. Models with insufficient fit (<0.75) and unrealistic fits (nearly linear) are denoted with - and *, respectively.

Trimetoprim	PFO			PSO		
	q_e	k_1	R^2	q_e	k_2	R^2
Horse manure 220	-	-	-	-	-	-
Sewage sludge 220	-	-	-	-	-	-
**Fluconazole**						
Horse manure 220	26.48	0.318	0.952	29.16	0.0154	0.959
Sewage sludge 220	-	-	-	-	-	-
**PFOA**						
Horse manure 220	-	-	-	-	-	-
Sewage sludge 220	-	-	-	-	-	-
**Arsenic**						
Horse manure 220	-	-	-	-	-	-
Sewage sludge 220	36.24*	0.000244*	0.892*	28.60*	0.0000109*	0.892*
**Copper**						
Horse manure 220	23.37	0.0259	0.967	-	-	-
Sewage sludge 220	66.91	0.0402	0.980	92.96	0.000346	0.976
**Zink**						
Horse manure 220	-	-	-	-	-	-
Sewage sludge 220	-	-	-	-	-	-

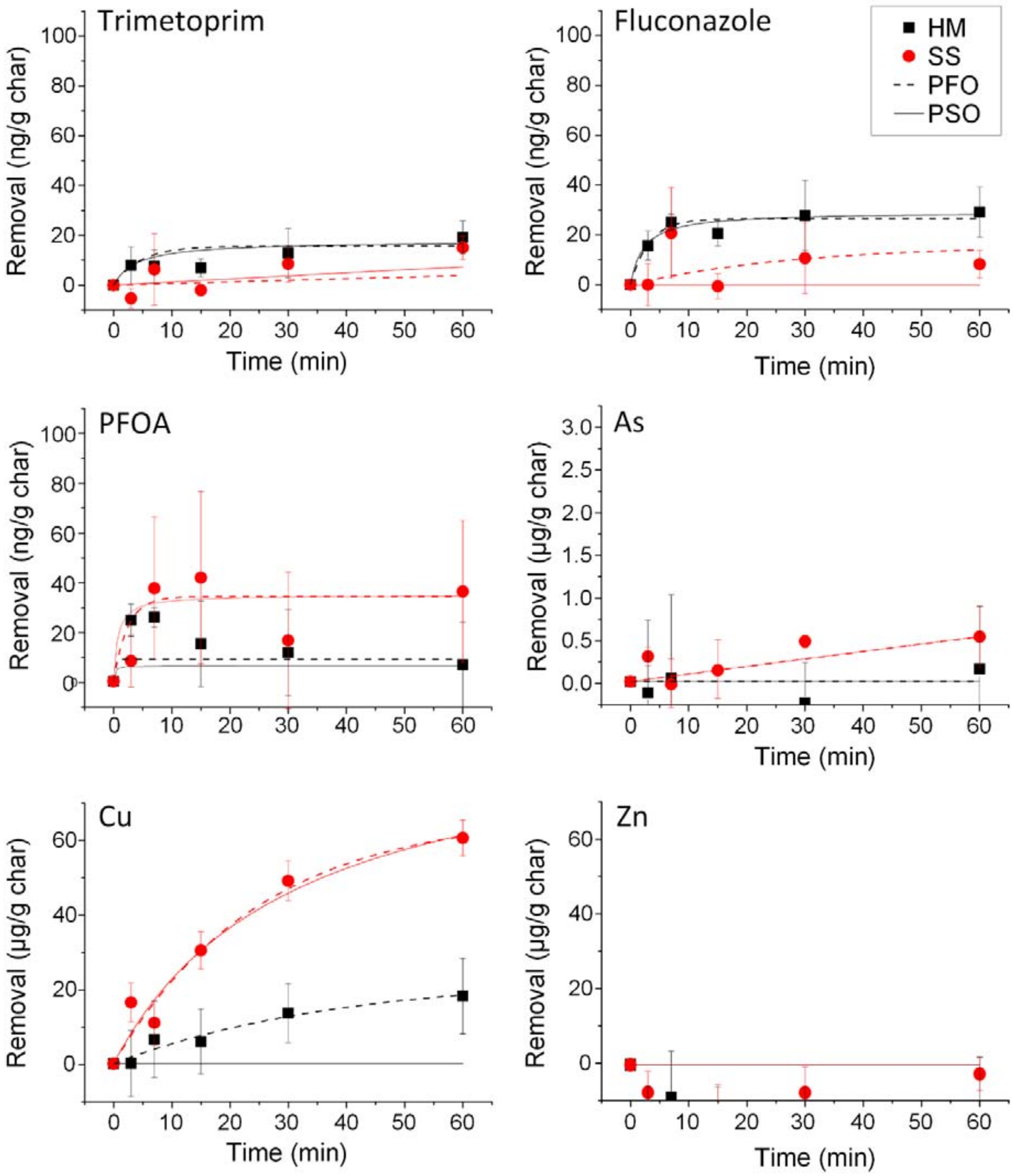

Figure S5. Removal kinetics of the studied compounds from ultrapure water on horse manure temperature series.
